# Markers of arterial health could serve as accurate non-invasive predictors of human biological and chronological age

**DOI:** 10.18632/aging.101227

**Published:** 2017-04-28

**Authors:** Alexander Fedintsev, Daria Kashtanova, Olga Tkacheva, Irina Strazhesko, Anna Kudryavtseva, Ancha Baranova, Alexey Moskalev

**Affiliations:** ^1^ Engelhardt Institute of Molecular Biology of Russian Academy of Sciences, Moscow 119991, Russia; ^2^ The Russian Clinical Research Center for Gerontology, Moscow 192226, Russia; ^3^ Lomonosov Moscow State University Medical Center, National Center for Preventive Medicine, Moscow 119234, Russia; ^4^ Moscow Institute of Physics and Technology, Dolgoprudny, Moscow Region 141700, Russia; ^5^ School of Systems Biology, George Mason University (GMU), Fairfax, VA 22030, USA; ^6^ Federal State Budgetary Institution “Research Centre for Medical Genetics”, Moscow 115478, Russia; ^7^ Atlas Biomed Group, Moscow 121069, Russia; ^8^ Institute of Biology, Komi Science Center of RAS, Syktyvkar 167982, Russia

**Keywords:** biological age, stenosis, pulse wave velocity, augmentation index, artery age

## Abstract

The decline in functional capacity is unavoidable consequence of the process of aging. While many anti-aging interventions have been proposed, clinical investigations into anti-aging medicine are limited by lack of reliable techniques for evaluating the rate of ageing. Here we present simple, accurate and cost-efficient techniques for estimation of human biological age, Male and Female Arterial Indices. We started with developing a model which accurately predicts chronological age. Using machine learning, we arrived on a set of four predictors, all of which reflect the functioning of the cardiovascular system. In Arterial Indices models, results of carotid artery duplex scan that show the thickness of the intima media complex and quantitatively describe the degree of stenosis are combined with pulse wave velocity and augmentation index measurements performed by applanation tonometry. In our cohort, the age of men was determined with MAE = 6.91 years (adjusted R-squared = 0.55), and the age of women with MAE = 5.87 years (adjusted R^2^ = 0.69). The Epsilon-accuracies of age-predicting models were at 86.5% and 80% for women and men, respectively. Substantially higher differences between the predicted age and the calendar age were noted for patients with Type 2 Diabetes Mellitus (T2D) as compared to non-T2D controls, indicating that the model could serve as a good approximation for an elusive biological age. Notably, in females with chronological and biological ages mismatching by 5 or more years, significant increases in in Framingham CVD scores and lower levels of IGF-1 were observed.

Proposed Male and Female Arterial Indices derive biological age from the results of functional tests which do not require specialized laboratory equipment and, therefore, could be performed in hospitals and community health clinics.

## INTRODUCTION

Measuring aging biologically rather than chronologically provides personalized view to an optimal, rather than “normal” or “typical” health. Throughout the course of life, each of us gradually departs from the health trajectory defined by our individual genome. Even in case of identical twins, substantial differences in the timing of the onset development of particular aging-associated symptoms are common-place. At later stages of ontogenesis, non-genetic factors, both exogenous and endogenous, gain in importance. Hence, adult individual's rate of ageing depends primarily on lifestyle rather than genes.

Newly introduced concept of anti-aging interventions enables individuals to actively modify their lifestyles or pharmacologically correct for accumulating bio-chemical or functional deficits. In order to properly evaluate relative efficiency of these interactions, objective measures of attained ageing are necessary. The quest for the best definition of biological age started in 1969, with the seminal paper of Comfort [1].

The chronological age is most easy to define as it typically can be traced to a birth record, while the quest to ascertain biological age without such a record still remains unattained. At best, biological age can be reflected by overall resemblance of an aged individual to an average degree of age-associated changes observed in a given population at given age. In the frame of this definition, any departure from population-wide standard of aging stems from a combination of environmental and genetic factors that either promote or delay the development and subsequent involution of various physiological systems and their capability to adapt. Therefore, a positive or a negative difference between biological and chronological age, observed in a given individual, may be interpreted as either speeding up or slowing down the ageing process, thus, providing a measure for an evaluation of one or another anti-ageing intervention.

There is a long history of attempts to determine biological age and quantify the tempo of the process of ageing. Typically, age determination utilizes one or another molecular facet of ageing, for example, the degree of the damage to cell's DNA [2]. Among more recently developed integrative biomarkers of aging is the GlycanAge index that profiles the structural details of sugar chains attached to the conserved N-glycosylation sites of three types of IgG molecules. This index reflects the level of systemic inflammation, predicts chronological age with standard deviation of 9.7 years, and is superior to age evaluation using telomere length [3]. Peripheral blood mononuclear cells (PBMCs) mRNAs-based “trans-criptome age” index predicts chronological age with mean absolute error of 7.8 years [4]. Even more precise PBMCs-based “epigenetic age” relies on quantitation of the methylation of three CpG sites located in *ITGA2B, ASPA* and *PDE4C* genes with standard deviation of less than 5 years [5]. An increase in the number of profiled CpG dinucleotides to 353 improves epigenetics-based age estimates by decreasing an error down to 2.9 years [6]. This technique is also capable of predicting mortality (p < 0.003) [7], but not the probability of major cardiovascular event [8]. It should be noted that all the techniques described above require specialized equipment and skilled laboratory personnel, thus, limiting their clinical applicability. On another end of the spectrum are age-predicting models not specifically connected to any particular mechanism of aging, for example, deep neural networks (DNNs) modules evaluating common blood biochemistry and cell count tests [9]. Though the accuracy of this model is quite high, the number of parameters in the model is also high. Since deep neural nets are, in a nutshell, “black boxes”, the dissection of these models into mechanistic insights into the process of ageing is impossible.

The majority of the techniques described above have not yet entered clinical practice. The major culprits causing this lack of translation to the clinic have been a high number of the parameters requiring evaluation, and the laboratory, rather than clinical nature of tests being performed. From clinical perspective, the most convenient estimate of biological age would be the one relying on a combination of biochemical and physio-logical parameters typically evaluated in course of annual physical exam. Recently, a number of attempts to derive biological age from readily available clinical parameters were published, including these developed using retrospectively analyzed NHANES [10], Dunedin Young Adults [11], Long Life Family Study [12] or a combination of large cohorts [13]. The latter study is especially interesting as it demonstrates an integrated multi-system physiological dysregulation, with no particular physiological system emerging as clear contender as a source of “sentinel” biomarkers [12].

In this study, we attempt the dissection of biochemical and clinical predictors of age, the development of the predictive model for biological age, and explore the deviation of these predictions from chronological age in a cohort of 303 individuals. We quantified 89 clinical and biochemical parameters, then selected the top five parameters with a highest Pearson's correlation with chronological age. Importantly, all five of these parameters reflect the functioning of the cardiovascular system. The outputs of the gender-specific linear regression models predicting chronological age were compared to actual age of the subjects. Substantially higher differences between the predicted age and the calendar age were noted for patients with Type 2 Diabetes Mellitus (T2D) as compared to non-T2D controls. We believe that the proposed gender-specific models, which we named Male and Female Arterial Indices, may serve as a good approximation for an elusive biological age. Importantly, the proposed age-approximation tech-niques rely on functional tests which do not require specialized laboratory equipment and, therefore, could be performed in hospitals and community healthcare settings.

**RESULTS**

Two separate age-predicting models were built for men and women, respectively. The model for predicting an age of women, so called Female Arterial Index (1), was fitted using 182 samples of training set. Mean age was 50 years, with a standard deviation of 12.4 years. None of participating women had hypertension or diabetes, only 18 female participants ever smoked.

$${\text{AGE}}_{W}=-59.92+48.87\cdot {\text{CIM}}_{\min}+2.4\cdot \text{AIx}+32.41\cdot \mathrm{PWV}+0.64\cdot {\text{STEN}}_{\max}-0.95\cdot \text{AIx}\cdot \text{PWV}-0.7\cdot {\text{CIM}}_{\min}\cdot {\text{STEN}}_{\max}$$ (1) ,

Where

*CIM_min_* – minimal thickness of the intima media complex, in the left or right carotid,
*AIx* – Augmentation Index (AIx),
*PWV* – pulse wave velocity,
*STEN_max_* – Maximal of two stenosis values, on the left or on the right.

**Figure 1:**
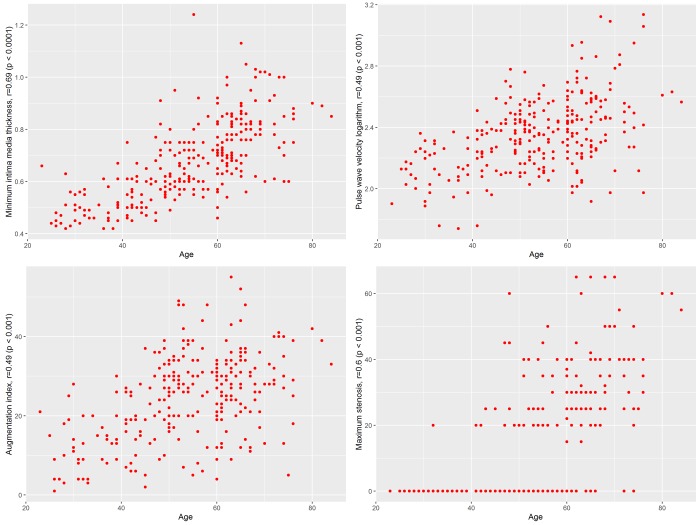
Correlation of the top predictors with age in female cohort

For each predictor, p-values are presented in Table 1, while correlations of each predictor with chronological age are shown in Figure 1. Female Arterial Index explains 69% of observed variation. Pearson's correlation coefficient of the model outputs with actual chronological age was at 0.8 (95% CI: 0.74-0.85). Standard deviation of residuals was 7.49 years, and MAE was 5.54 years.

**Table 1:** The statistical significance of each independent variable for the model predicting calendar age for females

	*CIM_min_*	*AIx*	*PWV*	*STEN_max_*	*STEN_max_* * *CIM_min_*	*AIx* * *PWV*
p-values for females	1.85e-14	0.003	0.00082	2.76e-06	0.012	6.68e-05

**Figure 2:**
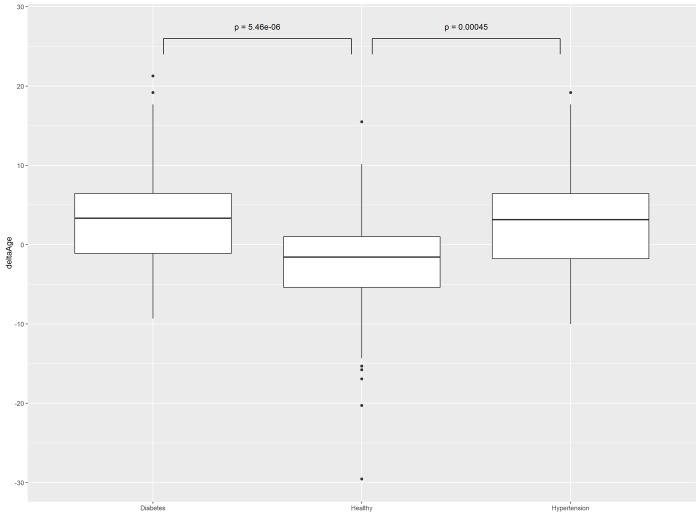
Differences between predicted age and chronological age in heath-stratified cohorts of women

To evaluate performance of the model, two groups of female subjects were enrolled. First group (n = 50, age = 55.4 ±7 years) included women with hypertension. In this cohort, 18 participants were smoking, a proportion significantly higher than that for training set (p < 0.001, chi-squared test). In a cohort of women with hyper-tension, Female Arterial Index performed with MAE of 5.68 years and epsilon-accuracy of 86%. Interestingly, in this cohort, the mean difference between predicted and actual age was 3.17 years, which was significantly (p <0.01) higher than mean difference observed in age-matched women subsample selected from training set (n = 118, age = 57.8 ±7 years, mean difference between predicted and chronological age = 0.02 years). In other words, according to Female Arterial Index, all women with hypertension were predicted to be, on average, three years older than healthier women of the training cohort (Fig. 2).

Second validation cohort (n=30, age = 56.5±5.64 years) included women with Type 2 diabetes (T2D). In this cohort, 17 participants were smoking, a proportion significantly higher than that in training set (p < 0.001, chi-squared test). In this validation set, Female Arterial Index performed with MAE of 6.06 years and epsilon-accuracy of 87%. The mean difference between predicted and chronological age was at 3.35 years, which was also significantly (p=5.46e^−06^) higher than mean difference for age-matched training set (Fig. 2).

Finally, when T2D and hypertension groups were joined, mean MAE for validation cohort was at 5.87 years with epsilon-accuracy of 86.5%.

Male Arterial Index (2) was developed for chronological age prediction in men. The model was fitted by training on the cohort of 95 male subjects, with mean age of 45.6 years, standard deviation of 11.6 years and no history of T2D or hypertension. Seventeen of these subject reported history of smoking. Since the sample size for men was smaller than that for women, list of inputted features was reduced by limiting the model to significant predictors only.

$${\text{AGE}}_{M}=-0.86+46.68\cdot {\text{CIM}}_{\min}+0.17\cdot {\text{STEN}}_{\max}+6.18\cdot \text{PWV}$$ (2)

**Figure 3:**
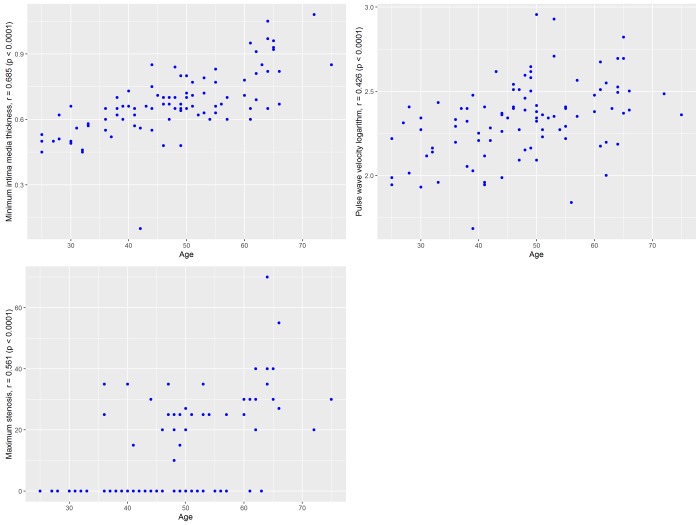
Correlation of predictors with male age

For each predictor, the statistical significance (Table 2), and the correlation with chronological age (Figure 3) were calculated.

**Table 2:** The statistical significance of independent variables inputted into Male Arterial Index predicting chronological age for males

	*CIM_min_*	*PWV*	*STEN_max_*
p-value for Men	0.012	0.045	0.0098

Male Arterial Index explains 54% of observed variation, with Pearson's correlation coefficient of its output with chronological age being at 0.73 (95% CI: 0.62-0.81)). Standard deviation of residuals was at 7.92 years and MAE was at 6.22 years.

**Figure 4:**
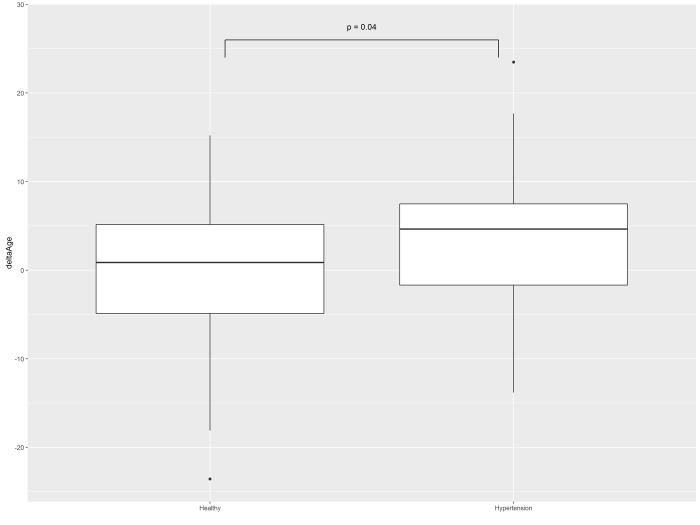
Differences between predicted age and chronological age in heath-stratified cohorts of men

For Male Arterial Index, the test set (n =30, age = 49.6 ±9.7 years) included subjects with hypertension. Among these, 17 subjects were smoking, which represents significantly larger proportion than that in training set (p < 0.001, chi-squared test). MAE for this test set was at 6.91 years, with epsilon-accuracy at 80%. The mean deviation of predicted and chronological age was at 4.03 years, significantly (p=0.02) higher than that for training set (−0.53 years) (Fig. 4).

To provide a benchmark for Male and Female Arterial Indices, Framingham CVD Prediction Scores were calculated [14]. For females (N=182), Framingham CVD Prediction Scores were correlated with both chro-nological age (r = 0.808, p-value < 2.2e^−^16) and Female Arterial Index values (r = 0.756, p-value < 2.2e^−16^).

When the CVD Prediction Scores were recalculated excluding age as an input, correlation with Female Arterial Age (r = 0.458, p-value = 2.803e-11) exceeded that with chronological age (r = 0.341, p-value = 2.197e-07). When females with more than five years of the difference between chronological age and biological age reflected by Arterial Index (N= 36) were compared to females with matching ages (N=145), statistically significant difference in their CVD Prediction Scores were noted (3.833 +/− 3.074 CVD Score units in age-mismatching group versus 2.31 +/− 2.987 CVD Score units in age-matching group, p-value = 0.005), thus indicating substantial health disparity in women with higher values of Arterial Index and, therefore, higher biological age. Interestingly, age-mismatching women also had lower levels of IGF-1 than age-matching controls (117.28 ng/ml +/− 52.66 ng/ml, mean = 101 ng/ml vs 150.299 ng/ml +/− 54.20 ng/ml, mean = 147 ng/ml; p-value < 0.001).

For men (N=95), correlations of Framingham CVD Prediction Scores with chronological age and with biological age approximated by Male Arterial Index were pronounced to substantially lesser degree (r=0.190, NS and 0.406, p-value < 3.655e^−05^, respectively). When men with more than five years of the difference between chronological age and biological age as reflected by Arterial Index (N=21) were compared to age-matching men (N=65), the differences in their CVD prediction scores had not reached statistical significance. Similarly, no significant differences in IGF-1 levels were noted.

In addition, Klemera-Doubal Biological Age estimates [10, 15] were calculated for males and for females, separately. For females, Klemera-Doubal model demonstrated MAE of 7.68 years with r2 = 0.352. The model correlated with chronological age with r=0.779 (p-value < 2.2e^−16^), and with biological age approximated by Female Arterial Index with r=0.969 (p-value < 2.2e-16). For males, Klemera-Doubal model demonstrated MAE of 8.46 years with r2 = 0.07. The model correlated with chronological age with r=0.696 (p-value < 8.25e^−12^), and with biological age approximated by Male Arterial Index with r=0.974 (p-value < 2.2e^−16^).

## DISCUSSION

The development of a simple, accurate and not expensive technique for biological age estimation is an important premise for evaluation of the anti-aging therapeutics or aging preventive medicines. Here we propose gender-specific Arterial Indices that reliably determine age by employing medical equipment which is common in hospitals and community-based clinics, with no molecular or cellular tests required. Arterial Indices evaluated non-invasively, by quantifying four functional indicators of cardiovascular health by a combination of a carotid artery duplex scan and applanation tonometry. In our cohort, the age of men was determined with MAE of 6.91 years (adjusted R-squared of 0.55), and the age of women with MAE of 5.87 years (adjusted R-squared of 0.69). Epsilon-accuracies were at 86.5% and 80% for women and men, respectively. Moreover, we showed that subjects with TD2M and hypertension have significantly higher difference between predicted and actual age than healthy controls. This observation indicates that our model could serve as an estimator of biological age. Interestingly, the proportions of smokers were larger in group with higher deviation of the predicted age and chronological age. These findings are consistent with previous observations of an association of smoking with both an acceleration of epigenetic ageing [16] and faster telomere loss [17].

Importantly, Arterial Indices evaluate biological age solely upon the parameters of cardiovascular health. The cardiovascular ageing is characterized by a series of complex pathophysiological changes affecting both the myocardium and the walls of the blood vessels at structural, cellular, molecular and functional levels. Heart and vessels conditions are the major component of age-related mortality [18]. Ageing is associated with functional changes in the blood vessels, including an increase in the stiffness of arteries that is the main contributor to hypertension [19]. Moreover, recent publication showed that an ageing of arteries correlates with chronological age much tighter than accompanying changes in biochemistry of the blood [20].

Carotid intima-media thickness (cIMT) is an established surrogate marker of atherosclerosis [21]. This parameter is also associated with metabolic syndrome, insulin sensitivity, and other age-related functional impairments [22, 23]. Moreover, intima-media thickness was shown to reliably predict the progression of Alzheimer's disease in general [24] and Alzheimer-associated cognitive decline in particular [25]. Moreover, revascularization improves cognitive function, thus, indicating that association between carotid artery stenosis and cognitive decline may be causative [26, 27]. In probands, cIMT values significantly correlate with parent's longevity, thus, highlighting a familial predisposition to be long-lived [28]. Moreover, cIMT is strongly and significantly associated with cardio-vascular and all-cause mortality [29].

Another cardiovascular predictor, Augmentation Index (AIx), is associated with the risk of symptomatic cardiovascular pathology development [30]. Aortic pulse wave velocity is a strong predictor of future CV events and all-cause mortality - an increase in aortic PWV by 1 m/s corresponded to an age-, sex-, and risk factor-adjusted risk increase of 14%, 15%, and 15% in total CV events, CV mortality, and all-cause mortality, respectively [31]. It is noteworthy that keeping cardiovascular health in a good shape leads to a reduction in the risk of cancer [32]. Recent study showed that the arterial age which is expressed as the degree of coronary artery calcification predicts an increased risk of cancer, chronic obstructive pulmonary disease, chronic kidney disease and hip fractures [33]. Senescent endothelial cells secrete microvesicles with miR-31 molecules, which inhibit osteogenic differen-tiation of mesenchymal stem cells [34]. In turn, an increase in arterial stiffness affects the oxygenation and the delivery of the nutrients to all other systems of human body. Thus, arterial aging could be seen as key contributor to an overall aging process.

Notably, individual components of the age-predictive Arterial Index are amenable to life style, dietary or medication-driven improvements. For example, gero-protective glucose-lowering compound acarbose were shown to decrease arterial stiffness by suppressing the levels of proinflammatory and profibrotic mediators CRP, PTX3, MMP-2 and MMP-9 in the plasma [35]. Pulse waive velocity may be improved by an increase in the intake of glutamic acid, leucine, and tyrosine [36], and by low dose valsartan intervention, either alone [37], or in combination with fluvastatin [38], or - in the mice models - with spermidine supplementation [39].

In our study, patients with more than five years of extra ageing implied by higher scores of Arterial Index also demonstrated significant elevation of CVD Prediction Scores [9] and lowered levels of serum IGF-1, the primary mediator of growth hormone (GH) effects, which is known to influence probability to develop major cardiovascular episode, all-cause mortality and cancer mortality [40-42] and diabetes [43]. While cardiovascular prognosis is reportedly affected by both low and high levels of IGF-1 [42], no consensus on optimal IGF-1 levels is reached. Paralleled measure-ments of IFG-1 levels and Arterial Indices may help in deciphering optimal mode of growth hormone signaling in various age groups.

We believe that the proposed Arterial Indices may be useful for the prediction of biological age, while the deviation of these predictions from chronological age may be treated as an indicator of an increase in arterial and, probably, overall rate of aging. Arterial Indices can be used either alone or in combination with other biomarkers of elusive biological age and serve as surrogate outcomes for the studies of anti-aging interventions.

## METHODS

For this study, we enrolled 450 subjects who visited the National Research Center for Preventive Medicine in Moscow, Russia, from May 2012 to December 2012. To determine eligibility, each subject completed a questionnaire on their medical history, a physical examination, and underwent a blood sampling. A total of 147 subjects were excluded based on current or previous history of coronary heart disease, peripheral arterial disease, arrhythmia, congestive heart failure, heart valve disease or stroke, hepatic or kidney failure, cancer diabetes, or current or past medication for diabetes, hypertension or hyperlipidemia. The chrono-logical age of the remaining 303 subjects varied from 23 to 91 year, with mean age of 52 +/− 13 years. In a group of 199 women, mean age was 53.87 +/− 13.58 years, with minimal age of 23 years and maximal age of 91 years. In a group of 104 men, mean age was 48.46 +/− 12 years, with minimal age of 25 years and maximum age of 75 year. For every patient, second assessment was performed in 12 months after the first one.

At inception, we analyzed 89 anthropometrical and biochemical parameters as well as a variety of the biomarkers of cardiovascular health, telomere length and the activity of telomerase. Below we present technical details for the parameters that received highest Importance scores.

### The medium intima thickness and the parameters of the carotid plaques

The carotid arteries were evaluated with high-resolution B-mode ultrasonography using linear hi-resolution probe of 17-5 MHz (PHILIPS iU22, Netherlands). All measurements were done by same operator. One longitudinal image of the common carotid artery and three longitudinal images of the internal carotid artery and the bifurcation were acquired in three projections (anterior, lateral, and posterior) and stored as 5 second loops. Additionally, the sectional images of all plaques were taken at the point of maximal vessel obstruction. The measurements of the carotid intima media thickness (cIMT) were performed in automatic mode. cIMT was assessed on the far wall of the common carotid artery, 1 cm proximal to the carotid bulb. All scans were measured by an experienced IMT reader using edge detection software. cIMTs on left and right sides were calculated as the maximal of nine distances between the carotid media–adventitia interface measured in three anterior, three posterior and three lateral projections, with an aid of 2DQ QLab software. cIMT(L)max and cIMT(R)max were equal to maximal cIMT measure-ment for the left and for the right carotid, respectively, while mean cIMT was further calculated as a sum of cIMT(L) and cIMT(R) divided by two.

For purposes of assessment, we have defined athero-genic plaque as a local thickening of the intima media complex by 1.5 mm, or a local thickening of the wall of the vessel by 50% as compared to its adjacent parts. All measurements were performed during diastole as defined by R-wave at simultaneously obtained cardiogram. After a statistical investigation of the strength of the associations between risk factors and intima–media thickness, the maximal rather than the mean intima–media thickness was selected as the key variable.

### Percent of the stenosis

The following angiographic parameters were obtained: minimal lumen diameter (millimeters), reference vessel diameter (millimeters), and percent diameter stenosis. The percent of the stenosis was calculated at the area of maximal narrowing of the vessel as the diameter of the remaining lumen (% stenosis (D)) and as the decrease in the open space in the vessel (% stenosis (S)). In this analysis, maximal values for % stenosis (D) and % stenosis (D) were taken into account after through study of all the carotid vessels. Additionally, a sum of all stenotic events observed in all carotic vessels was cal-culated as % stenosis (D)sum and и % stenosis (S)sum.

### Augmentation index and arterial stiffness

Augmentation index (AIx) was determined using the SphygmoCor 8.0 apparatus (PWV Medical, Sydney, Australia) and a high fidelity micromanometer. Data were collected after 20 sequential waveforms had been acquired, and averaged peripheral and corresponding central waveforms values calculated. The augmentation index was defined as the difference between the first (P1) and second (P2) peaks of the central arterial waveform, expressed as a percentage of the pulse pressure, and ejection duration as the time from the foot of the pressure wave to the incisura, as described previously [44].

Arterial stiffness was assessed according to the c-f PWV values with an aid of an applanation tonometer and electrocardiogram gating to attain pulse waves from both proximal (carotid artery) and distal (femoral artery) sites. The c-f PWV was calculated from the transit time between the two sites relative to the R-wave within the electrocardiogram complex using the ‘foot-to-foot method’ and the intersecting tangent algorithm [45]. For each subject, three sequential measurements were performed, and their mean value was considered for further analysis, after calculating the repeatability coefficient that was at 0.935.

### Statistical analysis

The most common problem in machine learning is overfitting, which occurs when number of predictors is high. To perform feature selection, Pearson coefficients of correlation were calculated for each parameter and chronological age. Table 3 lists all directly quantifiable measures, and their correlation with age (combinatorial measures are omitted). Five predictors with the highest values of Pearson correlation coefficient were subjected for further analysis. The values for pulse wave velocity (PWV) parameter were log-transformed.

**Table 3:** List of assessed clinical parameters ranged by their Pearson correlation coefficients

Clinical parameter	Correlation coefficient	p-value
Minimal of two cIMTs measured for left and right carotid (cIMTmin)	0.684	5.76e^−23^
Maximal of two Stenosis values (STEN_A)	0.596	2.04e^−22^
Central blood pressure (difference between systolic and diastolic)	0.505	6.52e^−22^
Augmentation Index (Aix)	0.481	3.90e^−13^
Pulse wave velocity (PWV)	0.456	2.23e^−12^
Total number of atherogenic plaques in left and right carotids	0.453	4.23e^−17^
B-type natriuretic peptide	0.364	0.0006
Von Willebrand factor	0.358	2.47e^−08^
IGF-1	">-([0-9]+)0.331	0.0004
C-peptide	0.324	0.0007
Telomere length	−0.319	0.015
Systolic Blood Presure	0.318	4.52e^−05^
Urea	0.269	6.74e^−05^
Presence of hypertension	0.264	0.0005
Presence of Diabetes	0.263	3.42e^−06^
Fibrinogen	0.258	0.01
Telomerase activity	−0.246	0.0016
Presence of obesity	0.245	0.012
Sodium	0.239	0.006
Glucose	0.228	0.0015
ESR	0.225	0.0025
Glycosylated hemoglobin	0.224	0.0015
Waist circumference	0.218	0.0022
BMI	0.194	0.0064
Hips ratio	0.193	0.0069
Cholesterol	0.188	0.01
HOMA IR	0.164	NS
Apolipoprotein A1	0.157	0.036
Waist to hip ratio	0.156	0.029
Apolipoprotein B	0.149	0.046
Insulin	0.146	NS
Insulin after 2h	0.141	NS
Potassium	0.131	NS
HDL	0.13	NS
LDL	0.125	NS
Creatinine	0.114	NS
Triglycerides	0.106	NS
Aldosterone	−0.104	NS

From clinical standpoint, models built upon easily interpretable regression are preferred to relatively laborious machine learning techniques, for example, Random Forest walk. Hence, for further reduction of the parameters we selected robust linear regression [46] implemented as a function lmrob in the software robustbase [47]. This type of regression models has an advantage of an easy interpretation coupled with lowered sensitivity to the outliers. To increase the sample size, for each patient we included the data collected both at study inception and at the follow-up, hence, representing a total of 606 sets of patients' descriptions (Females: n = 281; Males: n = 133). Adding interactions between the predictors significantly improved the model for women (Wald test, p < 0.0001), but failed to improve the performance of the model for men.

To measure the error of their respective models, a majority of previous studies use Root Mean Square Error (RMSE) which measures the average magnitude of the error, or the difference between a forecast and a corresponding observed values that are each squared and then averaged over the sample, followed by taking the square root of the average. Since the errors are squared before they are averaged, the RMSE gives a relatively high weight to larger errors. Due to this feature, RMSE recently became a subject of the critique for its sensitivity to outliers, and overestimation of the model error. To mitigate that, in current study, an accuracy of the final model was measured by Mean Absolute Error (MAE) which averages the absolute values of the differences between forecast and the corresponding observation. MAE is defined as:

$$\text{MAE}=\frac{1}{N}\sum_{i=1}^{N}|f_{i}-y_{i}|,$$

where *f_i_* is a prediction of the model, *y_i_* is a true value and N is a number of samples.

In addition, we also measured ε-prediction (epsilon-prediction) accuracy. In order to calculate epsilon-prediction accuracy, the sample is considered correctly recognized if the predicted age is in the range of [true age −ε; true age +ε]. In this study, we considered ε = 10. To check whether deltaAge parameters differ in groups of patients with hypertension and without this disease, and in groups with and without diabetes, Wilcoxon rank sum tests with continuity corrections were used. To correct for multiple comparisons, each p-value was adjusted using Holm's method.
